# Design and development of a sortilin‐targeting miniprotein for the treatment of FTD

**DOI:** 10.1002/alz70859_100961

**Published:** 2025-12-25

**Authors:** Moira Ek, Johan Nilvebrant, Per‐Åke Nygren, Stefan Ståhl, Hanna Lindberg, John Löfblom

**Affiliations:** ^1^ KTH Royal Institute of Technology, Stockholm, ‐ Sweden

## Abstract

**Background:**

Heterozygous loss‐of‐function mutations in the gene encoding progranulin (GRN) are causative in around 5‐10% of frontotemporal dementia (FTD) cases. These mutations lead to a more than 50% reduction of progranulin levels in the plasma and cerebrospinal fluid of mutation carriers compared to healthy controls. Increasing the progranulin levels in FTD‐GRN patients via inhibition of sortilin‐mediated progranulin degradation has shown promise as a therapeutic strategy, as exemplified by Alector’s latozinemab, currently in phase 3 clinical trials. Here we detail the development of an anti‐sortilin affibody‐based miniprotein as a non‐immunoglobulin alternative, offering potential advantages due to its small size and cost‐effective production.

**Method:**

Sortilin‐binding affibodies were selected by phage display and subsequently genetically fused to short peptides derived from the progranulin C‐terminus. The resulting miniproteins were characterized in terms of affinity, structure, stability, and their ability to increase extracellular progranulin levels in a clearance assay using progranulin‐secreting, sortilin‐expressing U‐251 MG cells.

**Result:**

A set of moderate‐affinity sortilin‐binding affibodies were obtained from phage display selections. Following genetic fusion with short peptides derived from the progranulin C‐terminus and optimization of the fusion constructs, a lead candidate with 185 pM affinity for sortilin was obtained. The affibody‐peptide fusion, but not its parental affibody or peptide, was capable of elevating extracellular progranulin levels in vitro with similar potency as latozinemab.

**Conclusion:**

A sortilin‐binding affibody‐based miniprotein was developed and optimized, performing on par with latozinemab in in vitro functional studies. Affibody‐based miniproteins provide a promising alternative to immunoglobulins in cases such as the present, where Fc functions are undesirable, and long‐term high‐dose antibody treatments risk becoming prohibitively expensive.

